# Indigo carmine: An organic crystal as a positive-electrode material for rechargeable sodium batteries

**DOI:** 10.1038/srep03650

**Published:** 2014-01-13

**Authors:** Masaru Yao, Kentaro Kuratani, Toshikatsu Kojima, Nobuhiko Takeichi, Hiroshi Senoh, Tetsu Kiyobayashi

**Affiliations:** 1 Research Institute for Ubiquitous Energy Devices, National Institute of Advanced Industrial Science and Technology (AIST) 1-8-31 Midorigaoka, Ikeda, Osaka 563-8577, Japan

## Abstract

Using sodium, instead of lithium, in rechargeable batteries is a way to circumvent the lithium's resource problem. The challenge is to find an electrode material that can reversibly undergo redox reactions in a sodium-electrolyte at the desired electrochemical potential. We proved that indigo carmine (IC, 5,5′-indigodisulfonic acid sodium salt) can work as a positive-electrode material in not only a lithium-, but also a sodium-electrolyte. The discharge capacity of the IC-electrode was ~100 mAh g^−1^ with a good cycle stability in either the Na or Li electrolyte, in which the average voltage was 1.8 V *vs.* Na^+^/Na and 2.2 V *vs.* Li^+^/Li, respectively. Two Na ions per IC are stored in the electrode during the discharge, testifying to the two-electron redox reaction. An X-ray diffraction analysis revealed a layer structure for the IC powder and the DFT calculation suggested the formation of a band-like structure in the crystal.

A change in using a rare metal (minor metal) in the current rechargeable battery systems is becoming a big challenge at the present time due to the increasing concern about resource problems and environmental burden. Rechargeable lithium batteries, which are widely used as power sources of various electronic devices, depend on rare metal oxides as the positive electrode active materials. Furthermore, lithium itself, an indispensable element as the carrier ion in rechargeable lithium batteries, is also a rare metallic element; therefore, the development of another battery system using a different carrier ion instead of the lithium ion is desired.

An alternative to lithium is to use sodium as the carrier ion in the rechargeable batteries^1^,^2^. So far, several types of positive-electrode active materials for use in the rechargeable sodium batteries have been proposed based on rare metal oxides (NaMO_2_; M = Co, Ni, Mn, or their mixture)^1^,^2^,^3^,^4^,^5^,^6^,^7^,^8^,^9^. Some efforts to get rid of rare metals have been made using iron-based materials (NaFeO_2_, NaFePO_4_, Na_2_FePO_4_F, prussian blue analogues)^10^,^11^,^12^,^13^,^14^; however, the variations in such electrode materials are still limited. One of the reasons for this limitation is ascribed to the larger ionic radius of the sodium ion, Na^+^, than that of the lithium ion, Li^+^. Namely, the large ionic size of sodium can impede its movement in the crystals of the active materials and/or cause severe structural changes in the active materials.

Based on this point, we developed the concept of using organic compounds instead of the conventional inorganic active materials for the sodium-based batteries. Many organic molecules, especially organic acids, form salts with both lithium and sodium ions, and some of them crystallize in similar motifs with either ion^15^,^16^,^17^. The volume changes due to the ion substitution from lithium to sodium are small compared with those of inorganic compounds. In addition, the preparation processes of such organic materials can be less energy demanding compared with those of inorganic materials because the latter are usually processed at higher temperatures than the former. The combination of organic materials that contain no scarce metal resources and a sodium-based electrolyte solution will realize a totally “rare metal-free” battery; however, the behaviors of such organic materials as the active materials of sodium battery systems are less well-known except for a few preceding examples^18^,^19^,^20^. Previously, we reported the battery performance of a series of redox active organic materials in lithium electrolyte systems^21^,^22^,^23^,^24^,^25^,^26^,^27^. In the present study, we prove that indigo carmine (IC, Fig. 1, R = SO_3_Na), a water-soluble organic molecule widely used as a food dye, can reversibly store not only lithium^22^, but also sodium via a redox reaction. The battery performance of IC as a positive electrode active material in a sodium electrolyte is described and the charge/discharge mechanism is discussed.

## Results

### Charge/discharge performance

Battery performance of the electrode using IC in a sodium system is compared to that in a lithium system^22^ in Fig. 2. In the sodium system, the IC electrode exhibited a discharge capacity of 106 mAh g_(IC)_^−1^ with an average potential of 1.8 V *vs.* Na^+^/Na for the first cycle (Fig. 2a). As proved in the next section, the positive electrode stores two Na ions per IC molecule during discharging. (N.B. Since two Na atoms in the sulfonate groups of IC are not involved in the redox reaction, one IC molecule holds a total four Na atoms in the reduced state.) The obtained capacity is quite close to the theoretical value of 115 mAh g_(IC)_^−1^ based on the assumption of a two-electron redox reaction per molecule as described in Fig. 2e. Meanwhile, although the charge/discharge curves in the sodium system have slightly more complex features than in the lithium system, the IC electrode behaved similar to the lithium system; the electrode showed a discharge capacity of 110 mAh g_(IC)_^−1^ with an average potential of 2.2 V *vs.* Li^+^/Li for the first cycle (Fig. 2b). The inflection point in the middle of the charge/discharge curves may imply the presence of an intermediate state of the IC molecule, such as a radical anion, at the half capacity. Similar inflections in the charge/discharge curves have been also observed in the electrodes made of the other organic molecules with multiple redox centers^21^,^23^,^25^,^26^. In many aqueous systems, the two-electron transfer to organic molecules directly takes place. If the electron-transfer to IC proceeds stepwise via radical anion, the phenomenon would be a characteristic feature of the non-aqueous systems. However, the detailed discussion is premature at present, because other interpretations of the inflection and plateau in the charge/discharge curve are possible, *e.g.*, a structural phase transition of the organic crystal and its biphasic coexistence. Most of the observed voltage differences between the sodium and lithium systems (0.4 V) can be explained by the redox potential difference (0.32 V) between lithium and sodium (*E*°_(Li^+^__/Li)_ = −3.03 V *vs.* NHE; *E*°_(Na^+^__/Na)_ = −2.71 V *vs.* NHE). This observation indicates that the insertion barrier of the sodium ion into the IC crystal is comparable to that of the lithium ion.

The cycling stability of the IC electrode in the sodium system is as good as in the lithium system (Figs. 2c and 2d). After 40 cycles, the discharge capacity in the sodium system dropped by 20 mAh g_(IC)_^−1^ from the initial capacity, which is very close to the capacity drop in the lithium system, 19 mAh g_(IC)_^−1^. In both the sodium and lithium systems, most of the capacity drop resulted only from the first cycle. The IC electrode became stable in the subsequent cycles with an almost 100% coulombic efficiency, suggesting a high reversibility of the IC crystal in storing Na^+^ or Li^+^. Nonetheless, a closer look at the cycling trend points to a slightly steeper capacity decay in the sodium system that may eventually result in a significant gap in the stability of the electrode between these two systems after long cycling. Furthermore, we confirmed a stable charge/discharge behavior of IC, even if the amount of the conductive additive is reduced to 25 wt% of the electrode (Supplementary Fig. S1).

The observed cycle-life performances are relatively good compared with the low-molecular-weight organic active materials reported for lithium systems which suffer from poor cycle performance due to the dissolution of the redox active molecules into the electrolyte solutions^23^,^24^,^25^,^26^,^27^. On the contrary, IC has a very low solubility in ordinary organic solvents due to the peripheral polar sulfonate groups. We consider that one of the reasons for the observed stable cycle-life in both lithium and sodium system is its low solubility in the electrolyte solutions, which should suppress the loss of the active material from the electrode during the charge/discharge process.

### Elucidation of the carrier ion and its stoichiometry

For the rechargeable lithium batteries, the carrier ion is typically the lithium ion; however, some organic positive-electrode active materials for lithium systems store and release anions, such as PF_6_^−^ or ClO_4_^−^, instead of Li^+^ during cycling^28^,^29^,^30^,^31^. Therefore, identifying the carrier ion is important in order to understand the charge/discharge mechanism. In the present study, we determined the charge carrier in the IC electrode in the sodium electrolyte to be Na^+^ and its stoichiometry using ion chromatography. Figure 3 plots the change in the stoichiometric ratio of the sodium ion to IC in the electrode, *R*_chr_(Na/IC), determined by ion chromatography versus the accumulated charge/discharge capacity, *C*^Σ^, observed electrochemically during the initial two cycles. The second *x*-axis represents the accumulated stoichiometric ratio of Na to IC, *R*^Σ^_el_(Na/IC), calculated from *C*^Σ^ under the assumption that all the electrochemical capacity was borne by the sodium ion transfer to IC; *i.e.*, *R*^Σ^_el_(Na/IC) = 3.6(*C*^Σ^/(mAh g^−1^))·(*M*_w_/(g mol^−1^))·(*F*/(C mol^−1^))^−1^, where *M*_w_ and *F* are the molecular weight and the Faraday constant, respectively. If this assumption is correct, the magnitude of the slope, |Δ*R*_chr_(Na/IC)/Δ*R*^Σ^_el_(Na/IC)|, should be unity. The slight increase in the background may imply side reactions involving Na^+^ at the interface of the electrode such as the formation of a surface layer during early cycling. However, the fact that the magnitude of the sloe is close to unity, |Δ*R*_chr_(Na/IC)/Δ*R*^Σ^_el_(Na/IC) = 0.92 ± 0.10 (Mean ± SD), corroborates that the charge carrier in this system is Na^+^.

### XRD analysis of the electrodes upon cycling

The changes in the crystal structure of IC in the electrode were analyzed using an ex-situ X-ray diffraction (XRD) measurement during the charge/discharge process. Figure 4 compares the XRD patterns of the IC powder, its initial electrode, and the ones after specific charge/discharge processes. During the first discharge, the XRD peaks ascribed to the charged structure became weak, and other broad peaks appeared at different angles. Such changes in the XRD patterns during the insertion process of a carrier ion is often observed for the electrodes using the crystalline organic active materials in the lithium systems^21^,^26^. By subsequent charging, the pattern of the electrode was restored to the original charged structure. A similar change was observed during the next cycle. This repeatable observation indicated that the changes in the crystal structure of IC via redox are reversible.

### Crystallographic structure analysis

**Figure 1:**
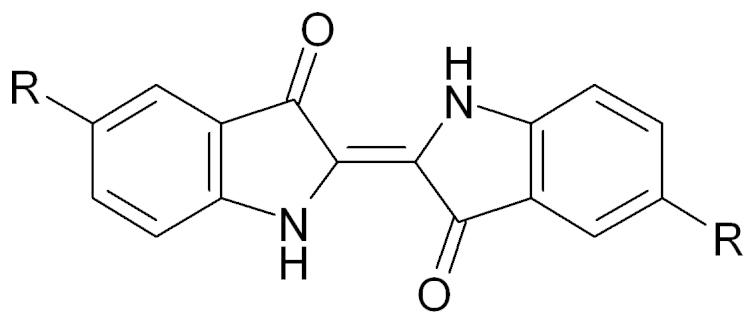
Chemical structure of indigo derivatives. R = H: indigo; R = SO_3_Na: indigo carmine (IC).

**Figure 2:**
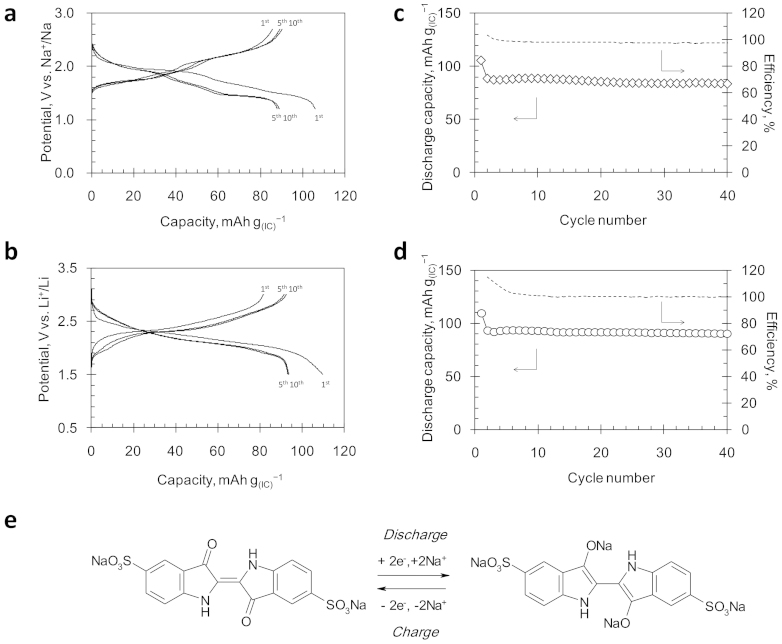
Charge/discharge performances of the IC electrodes. (a), Charge/discharge curves in the sodium system. Current density: 10 mA g^−1^, Potential range: 1.2–2.7 V *vs.* Na^+^/Na, Temperature: 30°C. (b), Charge/discharge curves in the lithium system. Current density: 10 mA g^−1^, Potential range: 1.5–3.0 V *vs.* Li^+^/Li, Temperature: 30°C. (c), Cycle-life performance in the sodium system. (d), Cycle-life performance in the lithium system^22^. (e), Two-electron redox reaction of IC in a sodium electrolyte system.

**Figure 3:**
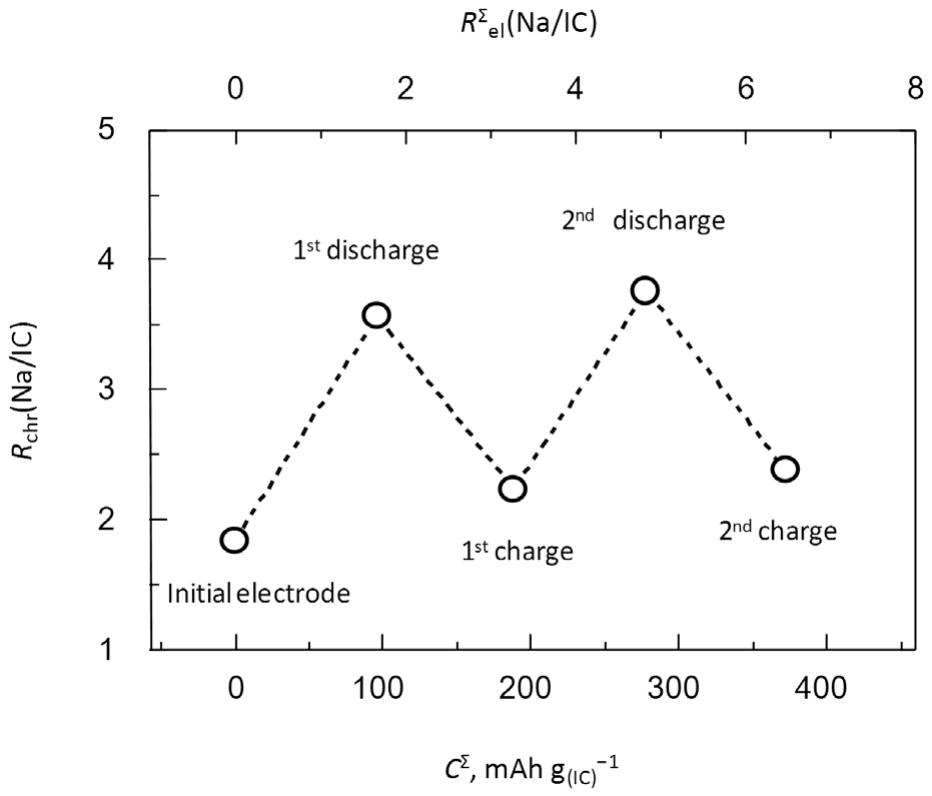
Stoichiometric ratio of Na to IC in the electrode, *R*_chr_(Na/IC), determined by ion chromatography versus the accumulated charge/discharge capacity, *C*^Σ^, determined electrochemically (initial two cycles). The second *x*-axis indicates the accumulated stoichiometric ratio of Na to IC, *R*^Σ^_el_(Na/IC), calculated from *C*^Σ^.

**Figure 4:**
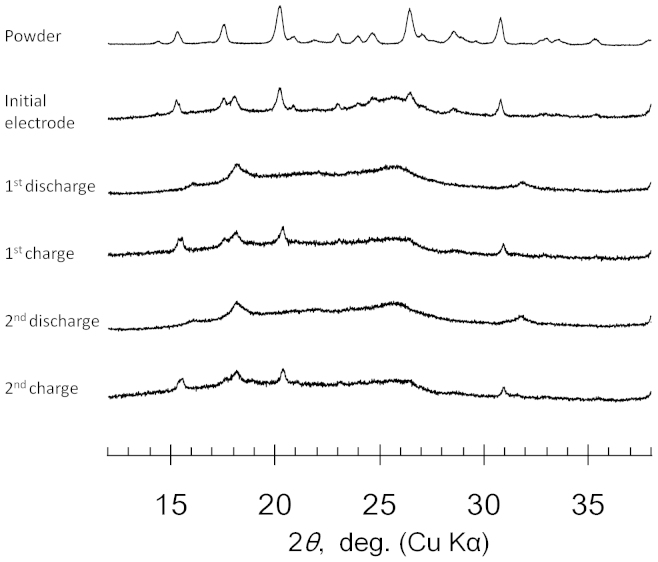
XRD patterns of the IC electrodes upon cycling. The peak at 18° and the broad peak at around 26° are ascribed to the polytetrafluoroethylene (PTFE) binder and the acetylene black (AB) conductive additive in the electrodes, respectively.

**Figure 5:**
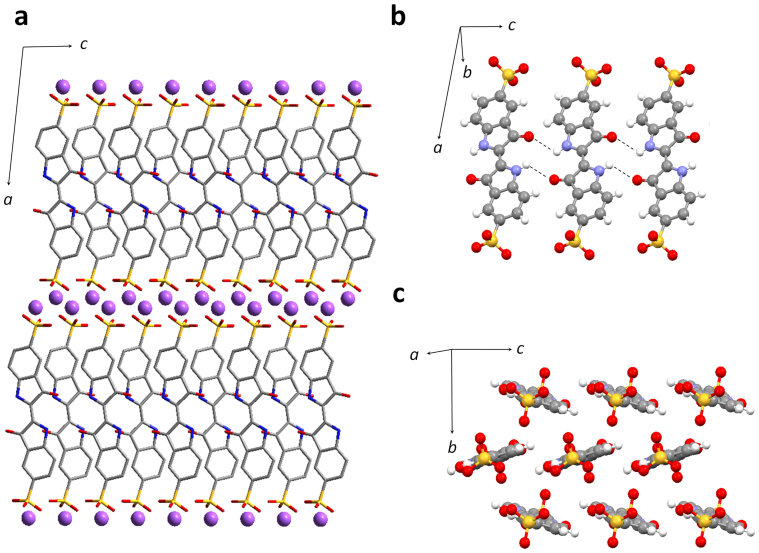
Crystal structure of IC (a polymorphic form crystallized in the present study). (a), Overview of the crystal. IC molecules and sodium ions are expressed using tubes and balls, respectively. Hydrogen atoms are omitted for clarity. (b), Hydrogen bonded layer composed of the IC molecules along the *c*-axis. (c), A two-dimensional packing of the molecules in the *bc*-plane. Sodium ions are omitted for clarity in (b) and (c). Crystallographic parameters, Formula: C_16_H_8_N_2_Na_2_O_8_S_2_, *M*: 466.353, Crystal system: monoclinic, Space group: *P*2_1_/*c*, *a*: 17.7145(6) Å, *b*: 8.0277(4) Å, *c*: 6.1637(2) Å, *β*: 97.287(2)°, *V*: 869.44(6) Å^3^, *Z*: 2, *T*: 298 K, *D*_calc_: 1.78 g cm^−3^, *R*_wp_: 6.01%, *S*: 1.17.

During the course of our research study of the organic active materials in the lithium electrolytes, we came to consider that how the molecules are aligned in the crystal plays a key role in understanding the charge/discharge mechanism^21^,^26^. The detailed crystallographic structure of IC has not yet been reported. During the recrystallization, we found that the IC molecule can be crystallized in a different polymorphic structure from the one that hitherto investigated. This polymorphic form allowed use to solve the crystallographic structure of IC using a direct space method^32^,^33^ followed by the Rietveld refinement^34^. Figure 5 represents the obtained crystal structure with the structural parameters derived from the refinements in the caption. The optimized parameters, *R*_wp_ and *S*, in the Rietveld refinement converged to reasonable values, resulting in a good agreement between the calculated and the experimental XRD profiles (Supplementary Fig. S2). As seen in Fig. 5a showing an overview image of the crystal structure, a layer composed of sodium ions and that of IC molecules are alternately stacked. Such layer structures are often seen for molecules containing sulfonate groups^35^. In the sodium layers, the sodium atoms are surrounded by several oxygen atoms of adjacent sulfonate groups of the IC molecules with the distances of about 2.4–2.6 Å. In the molecular layer, the IC molecules are bound one-dimensionally by hydrogen bonding between the oxygen atoms of the carbonyl groups and the hydrogen atoms of the imino groups of the neighboring molecules along the *c*-axis (Fig. 5b). In the IC crystal, the π-stacked columnar structure is absent (Fig. 5c), which is conspicuous in the indigo crystal, *i.e.*, R = H in Fig. 1 (Supplementary Fig. S3)^36^. The crystallograohic analysis of the original polymorphic form gave equivocal solutions (Supplementary Fig. S4); however, the similarity in the electrochemical properties between these two polymorphs (Supplementary Fig. S5) implies that the local structures do not significantly differ.

### Quantum chemical calculations

**Figure 6:**
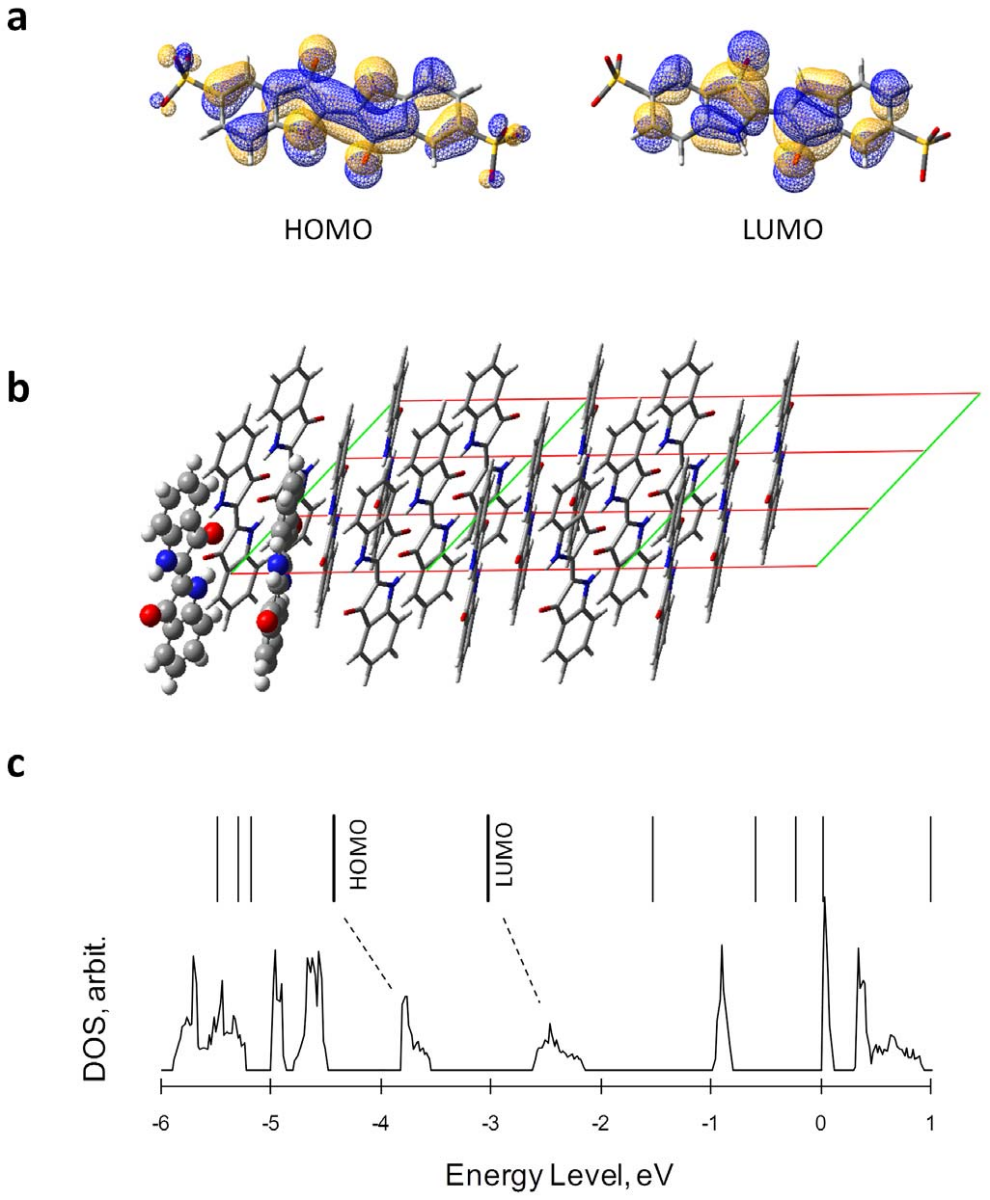
Quantum chemical calculations for IC. (a), Optimized structure of the monomer state of IC and the calculated highest occupied molecular orbital (HOMO) and lowest unoccupied molecular orbital (LUMO). Sodium ions are omitted for clarity. (b), Two-dimensionally aligned structure. In this model, the sulfonate groups were substituted by hydrogen atoms. (c), Calculated density of state (DOS) for the two-dimensionally aligned molecules along with the energy level of molecular orbitals of the monomer state. Computations were performed at the BLYP/6-31G (d) level. For the DOS calculation, the periodic boundary conditions (PBC) were taken into consideration.

To obtain a theoretical insight into the electronic conduction mechanism of the crystalline states of IC, the electronic structures were theoretically calculated based on the density functional theory. First, the calculated highest occupied molecular orbital (HOMO) and lowest unoccupied molecular orbital (LUMO) of the isolated IC molecule are shown in Fig. 6a. Each molecular orbital has π bond characteristics and is delocalized over the entire π-system of the molecule. Since IC undergoes reduction reactions during the discharge processes, the electronic state of the unoccupied orbitals, such as the LUMO, is important in order to accept electrons.

The electronic state of the molecular layer (Fig. 5c) was estimated under the periodic boundary condition (PBC) in which the peripheral sulfonate groups of the indigo skeleton were substituted by hydrogen atoms for simplification (Fig. 6b). The density of state (DOS) calculated for the molecular layer is shown in Fig. 6c along with the energy levels of the monomer orbitals. The energy levels of the many monomer orbitals expand, forming electronic band structures. The bands at around −3.7 and −2.4 eV originated from the HOMO and LUMO of the isolated monomer, respectively. The formation of such a band-like electronic structure indicates that adequate overlapping of the π-orbitals exists in the molecular layer of the crystal.

The electronic band structure can serve as electron-transfer pathways during the charge/discharge processes; thereby electrons can flow into the interior of the crystals, which is not directly attached to the current collector or a conductive additive. Although the mechanism of electronic conduction or ionic conduction in the crystals has not been experimentally revealed at present, the characteristic electronic structure should be related to the high utilization ratio of the IC crystal during the charge/discharge processes.

## Discussion

As proved by the charge/discharge profile, IC can work as a positive-electrode active material in both the lithium and sodium electrolyte systems. Furthermore, ion chromatography revealed that the IC molecule can store and release sodium ions as well as lithium ions accompanied by the redox reaction. We have previously found that a benzoquinone derivative worked as a positive-electrode material in electrolyte systems of not only sodium, but also divalent magnesium^37^. In general, organic molecules form their crystals by relatively weak intermolecular forces such as the van der Waals force, π-π interaction, and hydrogen bonding and the dimensionality of the packing motives often tends to be low. On the other hand, conventional inorganic active materials, such as metal oxides, form ionic crystals in which each atom is bound by a strong electrostatic force in three-dimensional manners. The organic molecular crystals are thought to be more flexible than the crystals of inorganic metal oxides from the viewpoints of the dimensionality of packing motif in the crystals and the differences in the interactions which bind each element. These characteristics are considered to make the electrochemical storage of large carrier ions possible. The high adaptability of such organic active materials for carrier ion species is a feature that is not often seen in typical inorganic active materials, indicating its usefulness for rechargeable sodium batteries. Furthermore, the electrochemical properties of such materials can be adjusted through molecular design, which would lead to further improvements.

## Methods

### Materials

Indigo carmine (5,5′-indigodisulfonic acid sodium salt, IC) as an active material was purchased from the Sigma-Aldrich Japan. The polymorphic crystalline powder was obtained by the recrystallization from the solution of a water/*N*-methylpyrrolidone system. Sodium bis(trifluoromethanesulfonyl)amide as an electrolyte salt and butylene carbonate as a solvent were purchased from Kishida Chemical and Tokyo Kasei, respectively.

### Preparation of electrodes and cells

For the battery tests, coin-type sealed cells were prepared as follows. A positive-electrode composite sheet was first prepared by mixing the IC powder, acetylene black (AB) as the conductive additive, and polytetrafluoroethylene (PTFE) as the binder in the weight ratio of 4:5:1. The sheet was then pressed onto an aluminum mesh current collector. The amount of active material deposited was approximately 3 mg per electrode. The prepared positive-electrode and a sodium metal negative-electrode were placed in an IEC R2032 coin-type cell case with a glass filter and a porous polypropylene separator (Celgard No. 2400, Celgard, Inc.). After the electrolyte solution (sodium bis(trifluoromethanesulfonyl)amide/butylene carbonate, 1.0 mol L^−1^) was added, the cell case was sealed. All the cells were prepared under a low humidity environment (dew point <−70°C).

### Measurements

For the charge/discharge cycle-life test, the prepared cell was galvanostatically discharged at the current density of 10 mA g_(IC)_^−1^ with a cut-off voltage of 1.2 V *vs.* Na^+^/Na, and charged at the same current density with a cut-off voltage of 2.7 V *vs.* Na^+^/Na using a charge/discharge system (ABE System, Electrofield) at 30°C. In this paper, the obtained capacities are expressed in terms of per mass of the active materials.

For the analysis of the indigo carmine crystal structure, powder X-ray diffraction (XRD) profiles were obtained using a diffractometer (SmartLab, Rigaku). Cu *K*_α1_ radiation (45 kV, 200 mA, *λ* = 1.540593 Å) monochromatized with a Ge(111) Johansson-type crystal monochromator was employed in the transmission method. The sample powders were contained in glass capillaries (0.5 mm diameter) and sealed with glue. The powder diffraction data were collected between 3 and 90° at 2*θ* with a 0.005 step. The obtained diffraction peaks were indexed by using the program N-TREOR^32^. The crystal structure was determined using the direct space method with the parallel tempering algorithm^33^ followed by the Rietveld refinement^34^. All the crystal structures were analyzed using integrated X-ray powder diffraction software (PDXL Version 2.1.1.4, Rigaku).

As for the analysis of the changes in the IC crystal structure of upon charge/discharge cycling, an ex-situ XRD measurement was applied using a diffractometer X'Pert PRO MPD (PANalytical). The electrodes removed from the cells after the charge/discharge cycling were washed with degassed tetrahydrofuran to dissolve out the electrolyte salt before the measurement.

The sodium ion concentration in the electrode was measured by an ion chromatography system (ICS-1000, DIONEX). The sodium concentration in the aqueous solution was determined in which the water-soluble contents were extracted by thoroughly immersing the electrode at a given state of charge and discharge.

### Theoretical calculations

A quantum chemistry calculation based on the density functional theory (DFT) was executed using the GAUSSIAN 03 program package. In order to estimate the electronic structure of the crystal state, a single point calculation considering the periodic boundary condition (PBC) was performed using the coordinates extracted from the X-ray analysis at the BLYP^38^,^39^/6-31G(d) level. The calculated molecular orbitals and the used coordinates were visualized by Gauss View 3.0.

## Author Contributions

M.Y. conceived the project, carried out the experiments, and prepared the manuscript. T.K. performed the ion chromatography. N.T. supported the crystallographic analysis. H.S. and K.K. assisted with the battery measurements and discussed the results. T.K. cooperated to advance this work and partially wrote the manuscript.

## Supplementary Material

Supplementary Information Supplementary Information
