# Supplementary material for: Indigo carmine: An organic crystal as a positive-electrode material for rechargeable sodium batteries
Source: Sci Rep. 2014 Jan 13;4:3650. doi: 10.1038/srep03650 (PMC3888987; doi:10.1038/srep03650)
Supplement: Supplementary Information [file srep03650-s1.pdf]

Supplementary Information

Indigo carmine: An organic crystal as a positive-electrode material for rechargeable sodium batteries

Masaru Yao*, Kentaro Kuratani, Toshikatsu Kojima, Nobuhiko Takeichi,

Hiroshi Senoh, and Tetsu Kiyobayashi

Research Institute for Ubiquitous Energy Devices,

National Institute of Advanced Industrial Science and Technology (AIST)

1-8-31 Midorigaoka, Ikeda, Osaka 563-8577, Japan

* Corresponding author;

Tel: +81-72-751-9651; Fax: +81-72-751-9629; E-mail: m.yao@aist.go.jp

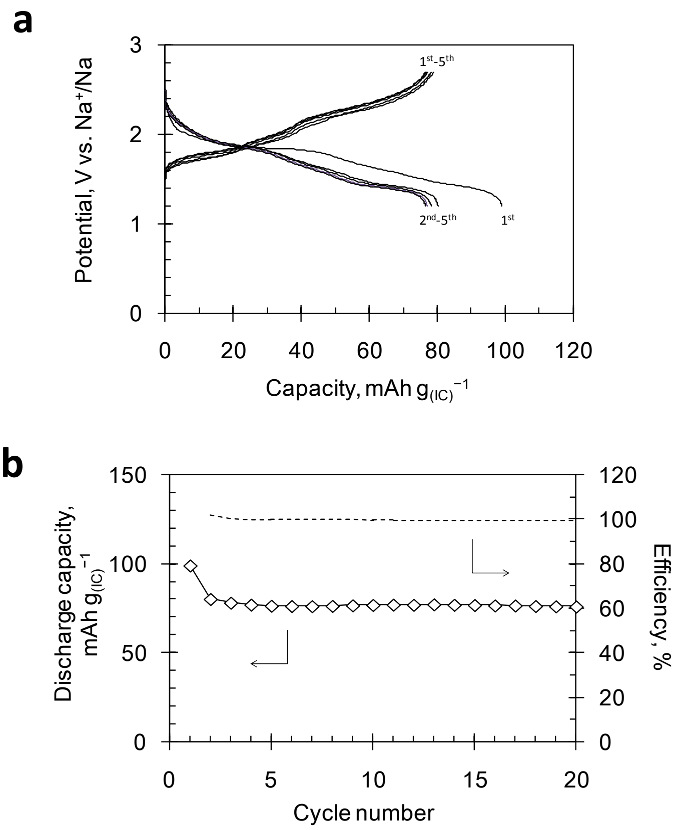

Figure S1 │Charge/discharge performance of a conductive-additive-reduced IC-electrode. a, Charge/discharge curves. **b,** Cycle-life performance. Composition: IC:AB:PTFE=70:25:5, Current density: 20 mA g^{-1}, Potential range: 1.2−2.7 V vs. Na^{+}/Na, Temperature: 30°C.

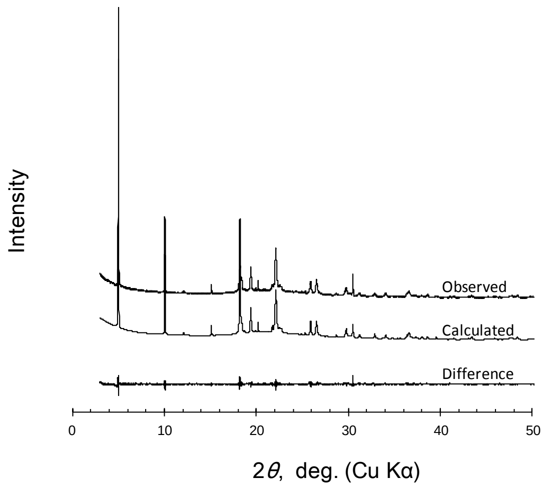

Figure S2 │ Powder XRD pattern and the profile fitting results of the polymorphic form of IC. Crystallographic parameters, Formula: $C_{16}H_8N_2Na_2O_8S_2$, *M*: 466.353, Crystal system: monoclinic, Space group: $P2_1/c$, *a*: 17.7145(6) Å, *b*: 8.0277(4) Å, *c*: 6.1637(2) Å, *β*: 97.287(2)°, *V*: 869.44(6) $Å^3$, *Z*: 2, *T*: 298 K, D_{calc}: 1.78 g cm^{-3}, R_{wp}: 6.01%, *S*: 1.17.

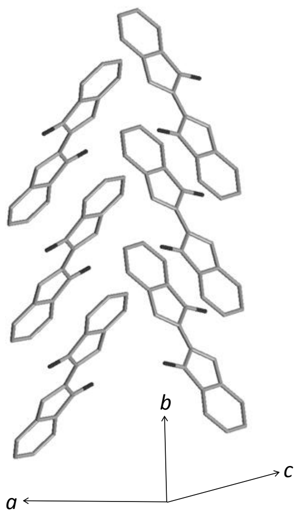

Figure S3 │ Crystal structure of indigo.[S1] Hydrogen atoms are omitted for clarity. Crystal structure data, Formula: $C_{16}H_{10}N_2O_2$, Crystal system: monoclinic, Space group: $P2_1/c$, a= 9.24 Å, b= 5.77 Å, c= 12.22 Å, β= 117.0°, Z=2, D_{calc}= 1.5 g cm^{-3}, R= 5.1 %.

Reference:

[S1] Süsse, P., Steins, M., & Kupcik, V. Indigo: crystal structure refinement based on synchrotron data. *Z. Kristallogr.* **184**, 269–273 (1988).

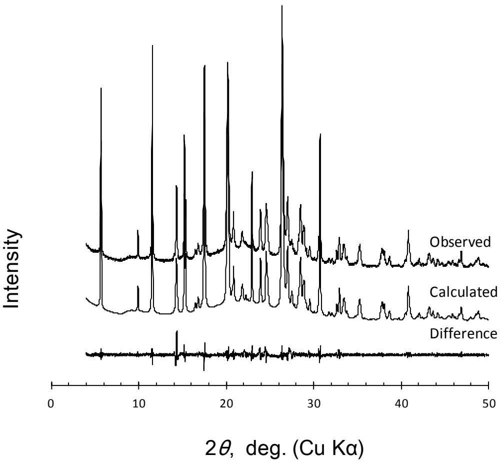

Figure S4 │ Powder XRD pattern and the profile fitting results of the original form of IC. Calculated pattern: crystal system: orthorhombic, a: 17.6 Å, b: 31.0 Å, c: 6.75 Å. In this analysis, we could not determine the space group. The assumed cell parameters might be wrong.

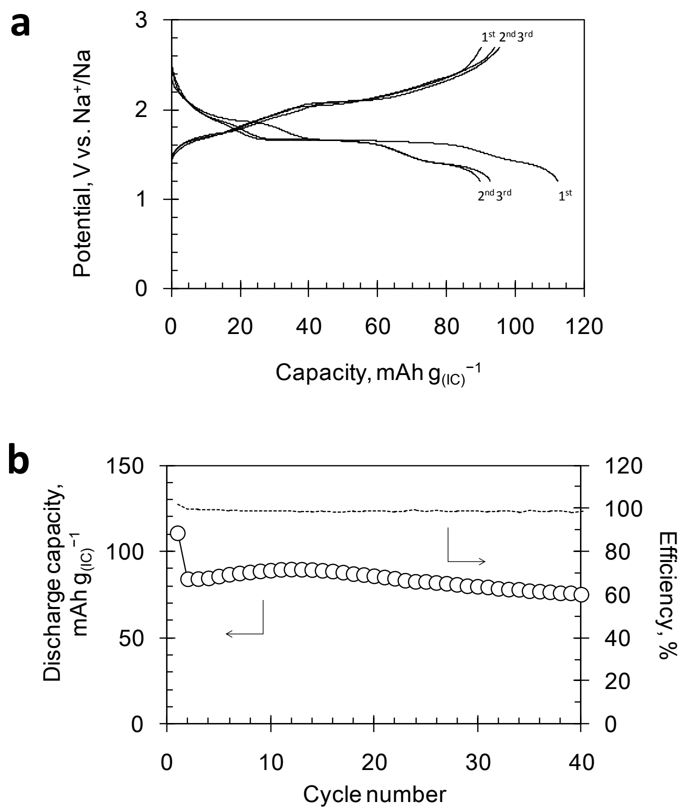

Figure S5│Charge/discharge performance of the electrode using the polymorphic form of IC in the sodium system. a, Charge/discharge curves in the sodium system. Current density: 10 mA g^{-1}, Temperature: 30°C. **b,** Cycle-life performance in the sodium system. Current density: 20 mA g^{-1}, Potential range: 1.2−2.7 V vs. Na^{+}/Na, Temperature: 30°C.

Crystallographic data (cif format)

```
#==============================================================================
data_global
#==============================================================================

_audit_creation_date                '2013-01-22'
_audit_creation_method              'PDXL Version 2.1.1.4 (Rigaku, 2012)'
loop_
    _audit_conform_dict_name
    _audit_conform_dict_version
    _audit_conform_dict_location
    cif_core.dic 2.4.2   ftp://ftp.iucr.org/pub/cif_core.dic
    cif_pd.dic     1.0.1   ftp://ftp.iucr.org/pub/cif_pd.dic

#==============================================================================
# PROCESSING SUMMARY (IUCr Office Use Only)

_journal_date_recd_electronic       ?
_journal_date_from_coeditor         ?
_journal_date_accepted              ?
_journal_coeditor_code              ?

#==============================================================================
# SUBMISSION DETAILS

_publ_contact_author_name           'Masaru YAO'
_publ_contact_author_address
;
Research Institute for Ubiquitous Energy Devices,
National Institute of Advanced Industrial Science and Technology (AIST)
1-8-31 Midorigaoka, Ikeda, Osaka 563-8577, Japan
;
_publ_contact_author_fax            '+81-72-751-9629'
_publ_contact_author_email          'm.yao@aist.go.jp'
_publ_contact_author_phone          '+81-72-751-9651'
_publ_contact_letter
;
    ENTER TEXT OF LETTER
;
_journal_name_full   'Scientific Reports'
_publ_requested_category            ?
_publ_requested_coeditor_name       ?

#==============================================================================
# TITLE AND AUTHOR LIST

_publ_section_title
;
Indigo carmine: An organic crystal as a positive-electrode material
for rechargeable sodium batteries
;
_publ_section_title_footnote
;
    ENTER FOOTNOTE TO TITLE OF PAPER
;
loop_
    _publ_author_name 'Masaru YAO'
    _publ_author_footnote
    _publ_author_address
;
    FIRST AUTHORS FOOTNOTES
;
;
    FIRST AUTHORS ADDRESS
;

#==============================================================================
# TEXT

_publ_section_synopsis
;
    ENTER SYNOPSIS
;
_publ_section_abstract
;
    ENTER ABSTRACT
;
_publ_section_comment
;
    ENTER TEXT
```

```
;
_publ_section_acknowledgements
;
        ENTER ACKNOWLEDGEMENTS
;
_publ_section_references
;
        ENTER OTHER REFERENCES

        PDXL Version 2.1.1.1 (2012).
        Integrated X-ray Powder Diffraction Software.
        Rigaku Corporation, Tokyo 196-8666, Japan.
;
_publ_section_figure_captions
;
        ENTER FIGURE CAPTIONS
;
_publ_section_exptl_prep
;
        ENTER COMPOUND PREPARATION DETAILS
;
_publ_section_exptl_refinement
;
        ENTER SPECIAL DETAILS OF THE REFINEMENT
;
#==============================================================================
data_IndigoCarmine
#==============================================================================
# CHEMICAL DATA

_chemical_name_systematic
;
5,5'-indigodisulfonic acid sodium salt
;
_chemical_name_common                'Indigo carmine'
_chemical_formula_structural         'C16 H8 N2 Na2 O8 S2'
_chemical_formula_moiety             '(Na +)2, C16 H8 N2 O8 S2 2-'
_chemical_formula_sum                'C16 H8 N2 Na2 O8 S2'
_chemical_formula_weight             466.353
_chemical_melting_point              ?
_chemical_compound_source            ?

#==============================================================================
# CRYSTAL DATA

_space_group_crystal_system          monoclinic
_space_group_name_H-M_alt            'P 1 21/c 1'
_space_group_name_Hall               '-P 2ybc'
loop_
        _symmetry_equiv_pos_site_id
        _symmetry_equiv_pos_as_xyz
        1    'x,y,z'
        2    'x,1/2-y,1/2+z'
        3    '-x,-y,-z'
        4    '-x,-1/2+y,-1/2-z'

_cell_length_a                       17.7145(6)
_cell_length_b                       8.0277(4)
_cell_length_c                       6.1637(2)
_cell_angle_alpha                    90
_cell_angle_beta                     97.287(2)
_cell_angle_gamma                    90
_cell_volume                         869.44(6)
_cell_formula_units_Z                2
_cell_measurement_temperature        298
_rigaku_reference_intensity_ratio    1.62
_pd_char_colour                      'dark-blue'
_exptl_crystal_density_diffrn        1.78
_exptl_crystal_F_000                 472.00

#==============================================================================
# EXPERIMENTAL DATA

_computing_data_collection           'Rigaku SmartLab'
_diffrn_measurement_device_type      'Rigaku SmartLabGuidance'
_diffrn_radiation_type               'Cu K¥a~1~'
_diffrn_radiation_wavelength         1.540593
_diffrn_measurement_method           2¥q
_diffrn_ambient_temperature          298

_pd_meas_2theta_range_min            3.0000
_pd_meas_2theta_range_max            90.0000
```

```
_pd_meas_2theta_range_inc                    0.0050
_pd_meas_number_of_points                17401

_reflns_number_total                     703
_reflns_limit_h_min                        0
_reflns_limit_h_max                       16
_reflns_limit_k_min                        0
_reflns_limit_k_max                        7
_reflns_limit_l_min                       -5
_reflns_limit_l_max                        5
_reflns_d_resolution_high                1.0902
_reflns_d_resolution_low                 17.5714

#==============================================================================
# REFINEMENT DATA

_computing_cell_refinement               'PDXL Version 2.1.1.4 (Rigaku, 2012)'
_computing_structure_solution            'PDXL Version 2.1.1.4 (Rigaku, 2012)'
_computing_structure_refinement          'PDXL Version 2.1.1.4 (Rigaku, 2012)'
_computing_publication_material          'PDXL Version 2.1.1.4 (Rigaku, 2012)'
_computing_molecular_graphics            '?'
_refine_special_details
;
;
_pd_calc_method                          'Rietveld Refinement'
_refine_ls_weighting_scheme              sigma
_refine_ls_structure_factor_coef         Fsqd
_refine_ls_hydrogen_treatment            mixed
_refine_ls_number_restraints             51
_refine_ls_number_constraints             1
_refine_ls_number_parameters             114
_refine_ls_matrix_type                   full
_pd_proc_ls_pref_orient_corr
;
    Spherical harmonics
    Maximum harmonic order: 6
;
_pd_proc_ls_prof_R_factor                0.0439
_pd_proc_ls_prof_wR_expected             0.0512
_pd_proc_ls_prof_wR_factor               0.0601
_refine_ls_goodness_of_fit_all           1.1736
_refine_ls_shift/su_max                  0.0190

loop_
    _atom_type_symbol
    _atom_type_description
    _atom_type_scat_dispersion_real
    _atom_type_scat_dispersion_imag
    _atom_type_scat_Cromer_Mann_a1
    _atom_type_scat_Cromer_Mann_b1
    _atom_type_scat_Cromer_Mann_a2
    _atom_type_scat_Cromer_Mann_b2
    _atom_type_scat_Cromer_Mann_a3
    _atom_type_scat_Cromer_Mann_b3
    _atom_type_scat_Cromer_Mann_a4
    _atom_type_scat_Cromer_Mann_b4
    _atom_type_scat_Cromer_Mann_c
    _atom_type_scat_source
    'C' 'C' 0.017 0.009
      2.31000       1.02000       1.58860       0.865000
     20.8439       10.2075        0.568700     51.6512        0.215600
;
    International Tables for Crystallography Vol. C, Table 6.1.1.4 (1999),
    and KEK Report 88-14, 1-136.
;
    'H' 'H' 0.000 0.000
      0.489918      0.262003      0.196767      0.049879
     20.6593        7.74039      49.5519        2.20159       0.001305
;
    International Tables for Crystallography Vol. C, Table 6.1.1.4 (1999).
;
    'N' 'N' 0.029 0.018
     12.2126        3.13220       2.01250       1.16630
      0.005700      9.89330      28.9975        0.582600    -11.529
;
    International Tables for Crystallography Vol. C, Table 6.1.1.4 (1999),
    and KEK Report 88-14, 1-136.
;
    'Na' 'Na' 0.130 0.124
      4.76260       3.17360       1.26740       1.11280
      3.28500       8.84220       0.313600    129.424        0.676000
;
```

```
        International Tables for Crystallography Vol. C, Table 6.1.1.4 (1999),
        and KEK Report 88-14, 1-136.
;
        'O' 'O' 0.046 0.032
            3.04850            2.28680            1.54630            0.867000
          13.2771              5.70110            0.323900         32.9089              0.250800
;
        International Tables for Crystallography Vol. C, Table 6.1.1.4 (1999),
        and KEK Report 88-14, 1-136.
;
        'S' 'S' 0.319 0.557
            6.90530            5.20340            1.43790            1.58630
            1.46790          22.2151              0.253600         56.1720              0.866900
;
        International Tables for Crystallography Vol. C, Table 6.1.1.4 (1999),
        and KEK Report 88-14, 1-136.
;

#===============================================================================
# ATOMIC COORDINATES AND DISPLACEMENT PARAMETERS

loop_
        _atom_site_label
        _atom_site_type_symbol
        _atom_site_fract_x
        _atom_site_fract_y
        _atom_site_fract_z
        _atom_site_U_iso_or_equiv
        _atom_site_adp_type
        _atom_site_occupancy
        S1    S    0.10270(12)    0.5129(5)     0.4123(5)     0.0110(13) Uiso 1
        Na1   Na   0.0231(3)      0.1820(8)     0.3697(10)    0.0110(13) Uiso 1
        O1    O    0.4182(2)      0.4320(11)    0.8128(9)     0.0110(13) Uiso 1
        O2    O    0.0602(3)      0.4418(9)     0.2201(8)     0.0110(13) Uiso 1
        O3    O    0.0797(3)      0.6809(7)     0.4488(12)    0.0110(13) Uiso 1
        O4    O    0.1021(3)      0.4088(9)     0.6042(9)     0.0110(13) Uiso 1
        N1    N    0.42578(19)    0.5487(8)     0.2681(9)     0.0110(13) Uiso 1
        C1    C    0.46231(16)    0.5063(9)     0.4700(10)    0.0110(13) Uiso 1
        C2    C    0.40404(14)    0.4741(8)     0.6171(8)     0.0110(13) Uiso 1
        C3    C    0.33187(14)    0.5008(6)     0.4836(7)     0.0110(13) Uiso 1
        C4    C    0.2576(2)      0.4886(7)     0.5279(7)     0.0110(13) Uiso 1
        C5    C    0.19954(17)    0.5235(6)     0.3622(7)     0.0110(13) Uiso 1
        C6    C    0.2174(2)      0.5680(8)     0.1555(8)     0.0110(13) Uiso 1
        C7    C    0.2913(2)      0.5792(9)     0.1084(8)     0.0110(13) Uiso 1
        C8    C    0.34807(15)    0.5460(7)     0.2738(7)     0.0110(13) Uiso 1
        H1    H    0.4477(3)      0.5728(11)    0.1555(12)    0.0110(13) Uiso 1
        H2    H    0.2468(3)      0.4598(9)     0.6669(10)    0.0110(13) Uiso 1
        H3    H    0.1780(3)      0.5909(11)    0.0454(11)    0.0110(13) Uiso 1
        H4    H    0.3020(3)      0.6094(13)   -0.0298(11)    0.0110(13) Uiso 1
#===============================================================================
# MOLECULAR GEOMETRY

_geom_special_details
;
        ENTER SPECIAL DETAILS OF THE MOLECULAR GEOMETRY
;
loop_
        _geom_bond_atom_site_label_1
        _geom_bond_atom_site_label_2
        _geom_bond_distance
        _geom_bond_publ_flag
        _geom_bond_site_symmetry_1
        _geom_bond_site_symmetry_2
        S1    O2    1.438(6) yes . .
        S1    O3    1.435(7) yes . .
        S1    O4    1.449(7) yes . .
        S1    C5    1.783(4) yes . .
        O1    C2    1.247(8) yes . .
        N1    C1    1.371(7) yes . .
        N1    C8    1.382(4) yes . .
        C1    C1    1.344(4) yes . 3_666
        C1    C2    1.481(6) yes . .
        C2    C3    1.446(4) yes . .
        C3    C4    1.381(5) yes . .
        C3    C8    1.407(6) yes . .
        C4    C5    1.383(5) yes . .
        C5    C6    1.398(7) yes . .
        C6    C7    1.379(6) yes . .
        C7    C8    1.365(6) yes . .
        N1    H1    0.859(9) no . .
        C4    H2    0.930(8) no . .
        C6    H3    0.929(7) no . .
```

```
        C7      H4      0.927(9) no . .
loop_
        _geom_angle_atom_site_label_1
        _geom_angle_atom_site_label_2
        _geom_angle_atom_site_label_3
        _geom_angle
        _geom_angle_publ_flag
        _geom_angle_site_symmetry_1
        _geom_angle_site_symmetry_2
        _geom_angle_site_symmetry_3
        S1      C5      C6      120.3(4) yes . . .
        S1      C5      C4      120.2(4) yes . . .
        O2      S1      O3      111.8(5) yes . . .
        O2      S1      O4      112.7(6) yes . . .
        O3      S1      O4      112.5(5) yes . . .
        O2      S1      C5      106.9(4) yes . . .
        O3      S1      C5      106.3(4) yes . . .
        O4      S1      C5      106.0(3) yes . . .
        O1      C2      C3      130.2(4) yes . . .
        O1      C2      C1      124.7(3) yes . . .
        N1      C1      C1      127.4(6) yes . . 3_666
        N1      C1      C2      108.3(3) yes . . .
        N1      C8      C3      110.3(4) yes . . .
        N1      C8      C7      128.3(6) yes . . .
        C1      N1      C8      109.3(5) yes . . .
        C2      C3      C4      132.3(5) yes . . .
        C2      C3      C8      107.0(3) yes . . .
        C4      C3      C8      120.7(4) yes . . .
        C3      C4      C5      118.5(5) yes . . .
        C4      C5      C6      119.5(4) yes . . .
        C5      C6      C7      122.6(5) yes . . .
        C6      C7      C8      117.3(5) yes . . .
        C1      C1      C2      124.3(6) yes 3_666 . .
        C1      C2      C3      105.1(5) yes . . .
        C3      C8      C7      121.3(4) yes . . .
        C8      N1      H1      125.3(8) no . . .
        C1      N1      H1      125.4(6) no . . .
        C5      C6      H3      118.7(7) no . . .
        C7      C6      H3      118.7(8) no . . .
        C6      C7      H4      121.3(7) no . . .
        C8      C7      H4      121.4(7) no . . .
        C3      C4      H2      120.8(6) no . . .
        C5      C4      H2      120.7(6) no . . .
```
